# Signature miRNAs in peripheral blood monocytes of patients with gastric or breast cancers

**DOI:** 10.1098/rsob.180051

**Published:** 2018-10-31

**Authors:** Le Shu, Zhe Wang, Qizhi Wang, Yumeng Wang, Xiaobo Zhang

**Affiliations:** 1 Laboratory for Marine Biology and Biotechnology of Qingdao National Laboratory for Marine Science and Technology and College of Life Sciences, Zhejiang University, Hangzhou 310058, People's Republic of China; 2 Department of Gastroenterology, Tongji Hospital, Tongji University School of Medicine, Shanghai 200065, People's Republic of China; 3 Department of Gastroenterology, The First Affiliated Hospital of Bengbu Medical College, Bengbu 233030, People's Republic of China; 4 Department of Gastroenterology, Chaohu Hospital of Anhui Medical University, Hefei, People's Republic of China

**Keywords:** peripheral blood monocytes, breast cancer, gastric cancer, small RNA sequencing, biomarkers

## Abstract

The dysregulation of microRNAs (miRNAs), key posttranscriptional regulators of gene expression, is closely associated with cancer development. However, the miRNAs of monocytes, important cells of tumour immunity, have not been extensively explored. In the present study, the differentially expressed miRNAs of blood monocytes derived from gastric and breast cancer patients and healthy donors were characterized. The results indicated that 74 miRNAs were upregulated and 46 miRNAs were downregulated in monocytes of patients with breast or gastric cancers compared with the healthy donors, suggesting that these 120 miRNAs from transformed monocytes were associated with cancers. The differentially expressed miRNAs, 38 of which were novel, were further validated using quantitative real-time PCR. As an example, the results showed that miR-150-5p downregulated the CCR2 expression in monocytes by targeting Notch 3, thus leading to the suppression of tumorigenesis. The target gene analysis showed that 36 of the 120 miRNAs targeted cancer-related genes. KEGG pathway analysis indicated that the cancer-associated miRNAs were involved in pathways related to cancers, such as the HIF-1 signalling and the mTOR signalling pathways. Thus, our study provided new clues to comprehensively understand the relationship between miRNAs and cancers.

## 1. Introduction

Patient mortalities due to breast or gastric cancers, the prevalent cancers worldwide, rank high among the most common deaths due to cancer [1,2]. By far, the prognosis of cancer is closely linked to diagnostic technology. Thus, searching for efficient biomarkers with high specificity is particularly significant for the efficient diagnosis as well as the treatment of cancer. As is reported, cancer pathogenesis has a close link with epigenetic modulation [3]. MicroRNAs (miRNAs), key epigenetic regulatory factors, play very important roles in cancer pathogenesis [4]. Furthermore, miRNAs are potential robust biomarkers for cancer diagnosis characterized by noninvasiveness and convenience of detection [5].

As major players in posttranscriptional regulation of gene expression, miRNAs, endogenous 20–24 nucleotide (nt) non-coding small RNAs, are involved in all kinds of biological processes, such as development, metabolism, cell proliferation and death, cancer and infectious diseases [6]. A growing number of reports indicate that miRNA expression patterns have become critical identifying criteria for the initiation and progression of tumours [7,8]. By controlling the expression of their target genes, miRNAs participate not only in tumour suppression but also in tumour promotion. The difference in miRNA expression profiles can be used to discriminate cancer patients from normal subjects [7]. It has been reported that a profile of five serum miRNAs (miR-1, miR-20a, miR-27a, miR-34 and miR-423-5p), whose expression levels are correlated with various tumour stages, can be a signature for gastric cancer diagnosis [9]. Even the survival rates of cancer patients can be predicted by a seven-miRNA signature (miR-10b, miR-21, miR-223, miR-338, let-7a, miR-30a-5p and miR-126), which is closely linked to relapse-free and overall survival among gastric cancer patients [10]. The circulating miR-195 is a breast cancer-specific miRNA, which can discriminate breast cancer from normal tissues and other cancers with a sensitivity of 88% and a specificity of 91% [11]. MiR-155, another biomarker of breast cancer, is highly upregulated in sera of breast cancer patients [12]. In this context, miRNAs are essential for the diagnosis and treatment of cancers.

It is well known that monocytes derived from progenitors in the bone marrow play important roles in the host innate immune response and inflammatory-related diseases. Monocytes usually traffic in blood vessels and peripheral tissues. Under favourable conditions, monocytes can migrate into tissues and further differentiate into macrophages and dendritic cells. However, it has been found that the peripheral blood monocytes can be recruited by tumours [13]. The recruited monocytes do not tend to differentiate into pro-inflammatory macrophages (referred to as M1-type macrophages) [14]. In contrast, they tend to differentiate into anti-inflammatory macrophages (referred to as M2-type macrophages), making a contribution to the progression of cancers [15]. The mediators and cellular effectors of inflammation are important constituents of the local tumour environment. In some types of cancers, an oncogenic change induces an inflammatory microenvironment that promotes the development of tumours [14]. The monocytes, which are main regulators of cancer inflammation, have an essential role in systemic inflammatory response to tumorous diseases. For example, some investigations have revealed that an absolute amount of monocytes can be a prognostic index of diffuse large B-cell lymphoma, a kind of malignant tumour that tends to relapse [16]. As reported, the monocytes from cancer patients display a significant decrease in the CCR5 level [17], indicating a close link between monocytes and cancers. It has been found that the lymphocyte-to-monocyte ratio (LMR) can be prognostic in haematologic neoplasia, and that the high-risk patients ranked based on the LMR do not benefit from adjuvant chemotherapy [18–20]. In breast cancer, an elevated LMR has been reported to be associated with a favourable prognosis for patients [18–20]. The monocytes can promote tumour angiogenesis in various murine tumour models [21]. It has been reported that murine TEMs, a subset of monocytes expressing Tie2, are more angiogenic than their Tie2-negative counterparts [22]. Therefore, it can be speculated that monocytes play critical roles in tumour progression. However, the signature miRNAs in monocytes of cancer patients have not been extensively explored.

In this study, the miRNAs of monocytes from patients with breast or gastric cancers and healthy donors were characterized to obtain the common signature miRNAs from different cancers. The results showed that 120 differentially expressed miRNAs were associated with cancers.

## 2. Material and methods

### 2.1. Patients and healthy donors

A total of 15 breast cancer patients, 12 gastric cancer patients and 13 healthy donors were recruited from The First Affiliated Hospital of Bengbu Medical College in 2014 (table 1). The donors, aged 45–65 years, accounted for 67%, 75% and 77% of the breast cancer patients, gastric cancer patients and healthy donors, respectively. The patients with gastric cancer and the healthy donors comprised approximately equal numbers of males and females (table 1). The patients with breast cancer were female (table 1). Blood samples were collected from the patients and healthy donors before any therapeutic process. The diagnoses of breast and gastric cancers were validated by histopathology or biopsy. This investigation was approved by The Clinical Research Ethics Committee of The First Affiliated Hospital of Bengbu Medical College.

**Table 1.** Sample information. TNM, tumour node metastasis; n.a., not applicable.

variables	patients with gastric cancer (*n* = 12)		patients with breast cancer (*n* = 15)		healthy donors (*n* = 13)	
average age (years)	58 ± 23.1		60.2 ± 19.2		56 ± 18.4	
sex	male	6	male	0	male	6
	female	6	female	15	female	7
TNM stage	I	5	I	8	n.a.	n.a.
	II	6	II	4	n.a.	n.a.
	III	1	III	3	n.a.	n.a.

### 2.2. Isolation of peripheral blood monocytes

Blood was layered on an equal volume of lymphocyte separation medium (Histopaque^®^-1077, Sigma-Aldrich, USA), followed by centrifugation for 30 min at 400***g*** (room temperature). Subsequently, the peripheral blood monocytes were collected from the interface and washed with isotonic phosphate-buffered saline (PBS). The monocytes were isolated by a flow cytometer. Briefly, the monocytes were resuspended in 100 µl of PBS at 10^6^ cells/sample. Then, 5 µl of mouse anti-human CD14 antibody (BD Pharmingen) was added. After incubation on ice for 30 min, the sample was washed three times with PBS at 4°C. The sample was resuspended in 500 µl of PBS and subjected to flow cytometry to isolate the monocytes.

### 2.3. miRNA sequencing and sequence analysis

Total RNAs were extracted from samples using the RNAiso Plus extraction kit (Takara, Japan) according to the manufacturer's instructions. The integrity of total RNAs was evaluated using an RNA 6000 Nano LabChip kit (Agilent Technologies, Palo Alto, CA, USA) with RIN number greater than 6.0. Then, RNAs were used to construct small RNA libraries and sequenced on an Illumina HiSeq 2500/2000 platform (Novogene Company, Beijing, China).

The raw data acquired from the high-throughput sequencing were collected and then filtered to remove the sequences of adapters, contaminated reads and poly A tails. The filtered sequences ranging from 18 to 35 nt in length, which were mapped to the human genome, were selected and subjected to a BLAST search in the sequences of Rfam (Rfam: http://www.sanger.ac.uk/software/Rfam) and the GenBank database (GenBank: http://www.ncbi.nlm.nih.gov/blast/Blast.cgi) to determine the non-coding RNAs (rRNAs, tRNAs, snRNAs and snoRNAs) and mRNAs. After subtracting the non-coding RNAs and mRNAs, the remaining filtered sequences were used to search for the known miRNAs in the miRbase 21.0 by disallowing mismatches.

All unannotated mapped sequences were analysed by the miREvo [23] and miRDeep2 software [24] for predicting novel miRNAs. The miREvo software predicts miRNAs based on miRNA homologues of multiple-species whole-genome alignments [20]. Based on miREvo analysis, miRDeep2 can predict novel miRNAs by evaluating the secondary structures, the DL1 cleavage sites and the minimum free energy of the target tags [21].

For the identification of differentially expressed miRNAs, the miRNA expression fold change between healthy donors and cancer patients was calculated with the formula: Fold change = log2 (healthy donors/cancer patients). Pearson's *χ*^2^ test was performed to evaluate the significant difference in miRNA expression levels between two selected samples. Fold change (≥2 or ≤−2) and *p*-value (≤0.01) were combined to determine the final miRNA expression significance.

### 2.4. Quantification of miRNA and mRNA by real-time PCR

To examine the expression of mRNA or miRNA, total RNAs were extracted from monocytes with the mirVanaP^™^ miRNA isolation kit according to the manufacturer's instructions (Ambion, USA). The reverse transcription reaction was conducted using the First Strand cDNA Synthesis Kit (Toyobo, Japan). The miRNA primers were designed as described before [25,26]. U6 RNA was used as a loading control. Quantitative real-time PCR was performed with sequence-specific miRNA primers or mRNA primers (*NLRP9*, 5′-GGAATCGGAAGTAAGGAAACA-3′ and 5′-CTGGAAGATAATGGAGTGGCA-3′; *MYBPH*, 5′-AAGTCTCGCTCAATAAA CCCT-3′ and 5′-CCTCATCATCGGCAACTCGTA-3′; *OLIG1*, 5′-TGCCCGACAGTCCCTTCCTCT-3′ and 5′-GCCTCCCTTCGCTCAGCTTCT-3′; *ICAM1*, 5′-CTTGCGGGTGACCTCCCCTTG-3′ and 5′-GCACTTTCCCACTGCCCATCG-3′; *CCR2*, 5′-AGAGGTCTCGGTTGGGTTGT-3′ and 5′-ATCATAACGTTCTGGGCACC-3′; *Notch3*, 5′-CGTGGCTTCTTTCTACTGTGC-3′ and 5′-CGTTCACCGGATTTGTGTCAC-3′; *β-actin*, 5′-AGCCTCGCCTTTGCCGA-3′ and 5′-CTGGTGCCTGGGGC G-3′). The PCR mixture (25 µl) consisted of 5 µl of SYBR^®^ Premix Ex Taq, 0.5 µl of 10 µM forward and reverse primers, and 100 ng of cDNA template. The PCR conditions were 95°C for 1 min, followed by 40 cycles at 95°C for 15 s and 60°C for 45 s.

### 2.5. Prediction of target genes and functional analysis of predicted target genes

To predict the target genes of miRNAs, the prediction programs miRanda (http://www.microrna.org/microrna/home.do), PITA (http://genie.Weizmann.ac.il/pubs/mir07/mir07_data.htmlhtml) and RNAhybrid (http://bibiserv.techfak.uni-bielefeld.de/rnahybrid/) were employed. miRanda software predicts the target genes by evaluating the base pairing and RNA thermostability. PITA starts by scanning the 3′-UTRs for potential miRNA targets. RNAhybrid software can predict the target genes by determining the most favourable hybridization sites between two sequences (miRNAs and target RNAs). The overlapping genes predicted by the three algorithms were considered the target genes of miRNAs.

The KEGG pathways enriched for potential target genes were determined using the KOBAS software [27]. The KEGG pathways represent comprehensive knowledge on the molecular interaction, reaction and networks for metabolism, genetic information processing, environment information processing, cellular processes, organismal systems, human diseases and drug development.

### 2.6. Cell culture and transfection of miRNA or siRNA

THP-1 human monocytes were maintained in RPMI 1640 medium (Invitrogen, USA) supplemented with 10% heat-inactivated fetal bovine serum at 37°C in a humidified atmosphere with 5% CO_2_.

The THP-1 cells at a density of 1 × 10^5^ cells/well were transfected with 100 nM of synthesized miR-150-5p (5′-UCUCCCAACCCUUGUACCAGUG-3′), miR-150-5p-scrambled (5′-CACUGGUACAAGGGUUGGGAGA-3′), Notch3-siRNA (5′-GUCAAUGUUCACUUCGCAGUU-3′) or Notch3-siRNA-scrambled (5′-UUCUCCGAACGUGUCACGUTT-3′) using the Lipofectamine RNAiMax transfection reagent (Life Technology, USA) according to the manufacturer's manual. At 24 h after transfection, the cells were collected for later use. All miRNAs and siRNAs were synthesized by Shanghai GenePharma Co., Ltd (Shanghai, China).

### 2.7. Dual-luciferase reporter assay

The miR-150-5p-binding sites of the Notch3 3′-UTR (5′-GACGC…GAGTGTTG GGAGCCTCC…CACTC-3′) and the Notch3 3′-UTR mutant (5′-GACGC…GAGTCAACCCTCCCTCC…CACTC-3′) were cloned into the pmirGLO Dual-Luciferase miRNA Target Expression Vector (Promega, USA) and confirmed by DNA sequencing. Then, 50 nM of miR-150-5p or the control miRNA was co-transfected with 0.1 mg of Notch3 3′-UTR plasmid or Notch3 3′-UTR mutant plasmid into THP-1 cells using Lipofectamine 2000. At 36 h after co-transfection, the luciferase activity of cells was measured using the dual luciferase reporter assay system (Promega, USA) according to the manufacturer's protocol.

### 2.8. Statistical analysis

The numerical data were presented as the mean ± standard deviation. The data were processed using ANOVA. Student's *t*-test was employed to assess the significant difference between treatments. All assays were biologically repeated three times.

## 3. Results

### 3.1. Sequence analysis of miRNAs in the peripheral blood monocytes of cancer patients

To reveal the miRNAs associated with cancers, the miRNAs in the peripheral blood monocytes of breast or gastric cancer patients and healthy donors were sequenced. The sequencing analysis generated 14 045 164, 12 610 606 and 14 096 347 high-quality reads for monocytes derived from breast cancer patients, gastric cancer patients and healthy donors, respectively. The percentages of high-quality reads in the corresponding small RNA libraries were more than 98%. After removal of mRNA, rRNA, tRNA, snRNA and snoRNA sequences, a total of 11 051 085, 9 558 267 and 12 218 332 raw reads were obtained for the breast cancer, the gastric cancer and the healthy donor samples, respectively (table 2).

**Table 2.** Sequence analysis of miRNAs in the peripheral blood monocytes of cancer patients.

type	healthy donor	breast cancer	gastric cancer	total
raw reads	12 218 332	11 051 085	9 558 267	32 827 684
mapped reads	7 837 095	6 059 967	4 649 985	18 547 047
unique mapped reads	41 755	47 840	53 252	
known microRNAs reads	7 257 356	5 764 819	4 315 633	
unique known microRNAs reads	993	978	1004	

Based on the alignment analysis, a total of 7 837 095, 6 059 967 and 4 649 985 raw reads from the healthy donor sample, the breast cancer sample and the gastric cancer sample were mapped to the human genome sequences or miRBase 21.0 sequences, respectively (table 2). The reads of known miRNAs accounted for 92.60%, 95.12% and 92.80% in the mapped reads of samples from healthy donors, breast cancer patients and gastric cancer patients, respectively. The analysis indicated that the numbers of unique known miRNAs were 993 for the healthy donor, 978 for the breast cancer and 1004 for the gastric cancer samples (table 2).

### 3.2. Potential novel miRNAs

To identify novel miRNAs, the unannotated small RNAs were further analysed with the miREvo and miRDeep2 programs. A total of 38 novel mature miRNAs were revealed. Among them, 26, 27 and 29 miRNAs were obtained from the breast cancer, the gastric cancer and the healthy donor samples, respectively (table 3). The novel miRNAs ranged mainly from 18 nt to 24 nt in length (table 3).

**Table 3.** Potential candidates of novel miRNAs. The novel miRNAs were predicted by the miREvo [23] and miRDeep2 [24] software against the identified human miRNAs in the database (www.mirbase.org).

name	length (nt)	sequence (5′–3′)	genomic location
hsa-miR-309	22	ugacuacuuuguuugauuuugu	chr6:141 987 409 to 141 987 520: (−)
hsa-miR-6377	22	agguaacuagaaaacaaaugaa	chr11:6 094 850 to 6 094 961: (−)
hsa-miR-7686	21	aacuguuacagugccuggaac	chr15:39 428 118 to 39 428 228: (+)
hsa-miR-1031	22	auuuaggccuguauaaucaugg	chrX:66 067 715 to 66 067 825: (+)
hsa-miR-238	22	uagagcuccgaucccauccacc	chr1:149 928 372 to 149 928 483: (−)
hsa-miR-787	23	ugaauaguuuuaguaacucuuga	chr12:21 518 649 to 21 518 759: (+)
hsa-miR-5118	22	agccucccagucuggccugagu	chr1:55 318 653 to 55 318 764: (+)
hsa-miR-6984	22	aacaaauacaggaaagaguucu	chr10:31 731 261 to 31 731 369: (+)
hsa-miR-344	22	acaugcgcccucggcuucuggc	chr17:45 317 326 to 45 317 437: (+)
hsa-miR-995	22	aaaugaaucauguugggccugu	chr10:113 291 611..113 291 720: (−)
hsa-miR-6959	24	gugugugcaccugugucugucugu	chr19:18 284 663 to 18 284 774: (+)
hsa-miR-7222	20	ugaaggcucugagccgggag	chr10:78 069 730 to 78 069 840: (+)
hsa-miR-5126	22	cuccucgccccuucccccgcca	chr9:129 413 288 to 129 413 401: (−)
hsa-miR-6999	22	cuccugcccuccuugcuguaga	chr1:26 554 505 to 26 554 616: (+)
hsa-miR-5696a	22	ucagacuaccuaaaugagcacu	chr2:101 309 444 to 101 309 555: (+)
hsa-miR-339a	23	ucccuguccuccaggagcucacc	chr22:32 348 115 to 32 348 227: (−)
hsa-miR-1820	22	aucaucuaugaacacugaaguu	chr2:85 708 551 to 85 708 661: (−)
hsa-miR-288	22	uaguuuaugucgauugcaauuu	chr13:31 804 788 to 31 804 899: (−)
hsa-miR-76	22	agaaucuguugguaaagccucu	chr1:221 813 933 to 221 814 044: (−)
hsa-miR-6417	22	uuuauuucaaaggacagcugga	chr2:229 330 703 to 229 330 814: (−)
hsa-miR-788	21	augugaaauggaauauagaaa	chr2:171 749 310 to 171 749 420: (+)
hsa-miR-5107	19	cccuguucuucucugggcg	chr14:75 334 218 to 75 334 326: (−)
hsa-miR-959	18	aucugugggauuaugacc	chr8:46 040 231 to 46 040 338: (−)
hsa-miR-4940	21	uugcugaacaaaucuuauuuc	chr12:31 891 035 to 31 891 144: (+)
hsa-miR-7117	18	aucucggacgagccccca	chr6:84 968 003 to 84 968 110: (+)
hsa-miR-6917	22	uucguuucaggugggaggagaa	chr7:36 737 228 to 36 737 338: (+)
hsa-miR-880	20	guuuuaucugaggggaugga	chr11:94 184 641 to 94 184 751: (+)
hsa-miR-7075	22	agaagacaugguccugaaacug	chr20:5 653 389 to 5 653 500: (+)
hsa-miR-9903-1	22	uuauccuccaguagacuaggga	chr8:98 393 647 to 98 393 758: (−)
hsa-miR-423a	18	guggacggugugaggcca	chr19:24 003 099 to 24 003 206: (−)
hsa-miR-7051	23	uuguaucaguggcuuuaauucgu	chr3:154 718 040 to 154 718 151: (−)
hsa-miR-7044	18	uccccggcaucaccacca	chr12:130 010 031 to 130 010 138: (+)
hsa-miR-6546	22	cacgcucgagcucggaggcugu	chr17:15 944 814 to 15 944 925: (−)
hsa-miR-9899a	24	cgggccccgggcccucgaccggac	chr21:8 436 281 to 8 436 394: (+)
hsa-miR-1898	25	ccccccugcuaaauuugacuggcua	chr7:138 415 477 to 138 415 586: (+)
hsa-miR-463	21	cugggauuguccaucaugaug	chr8:133 920 297 to 133 920 407: (+)
hsa-miR-307	21	ucacccggggugugcucgacu	chr2:131 093 672 to 131 093 782: (−)
hsa-miR-5107	22	ugucucugccucaccuuccagu	chr1:223 762 095 to 223 762 206: (+)

Mature miRNAs are generated by Dicer digestion. Therefore, the specificity of the Dicer enzyme cutting site relates to a strong bias in the first position of possessed miRNA [28,29]. Generally, the first nucleotide of a miRNA tends to be U or A. The quality of sequencing data can be evaluated by assessing the 5′-bias of miRNAs. In this study, the results showed that 5′-U and 5′-A occurred most frequently in 38 novel miRNAs, which composed more than 60% of the total novel miRNAs (table 3). Thus, the novel miRNAs were reliable.

### 3.3. miRNAs of monocytes associated with cancers

**Figure 1.**
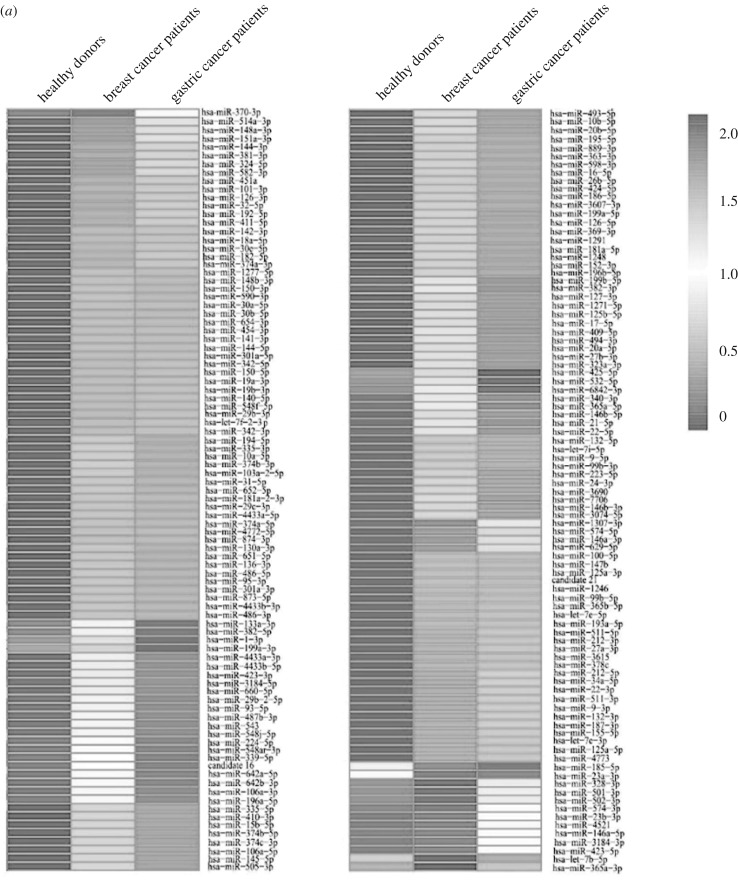

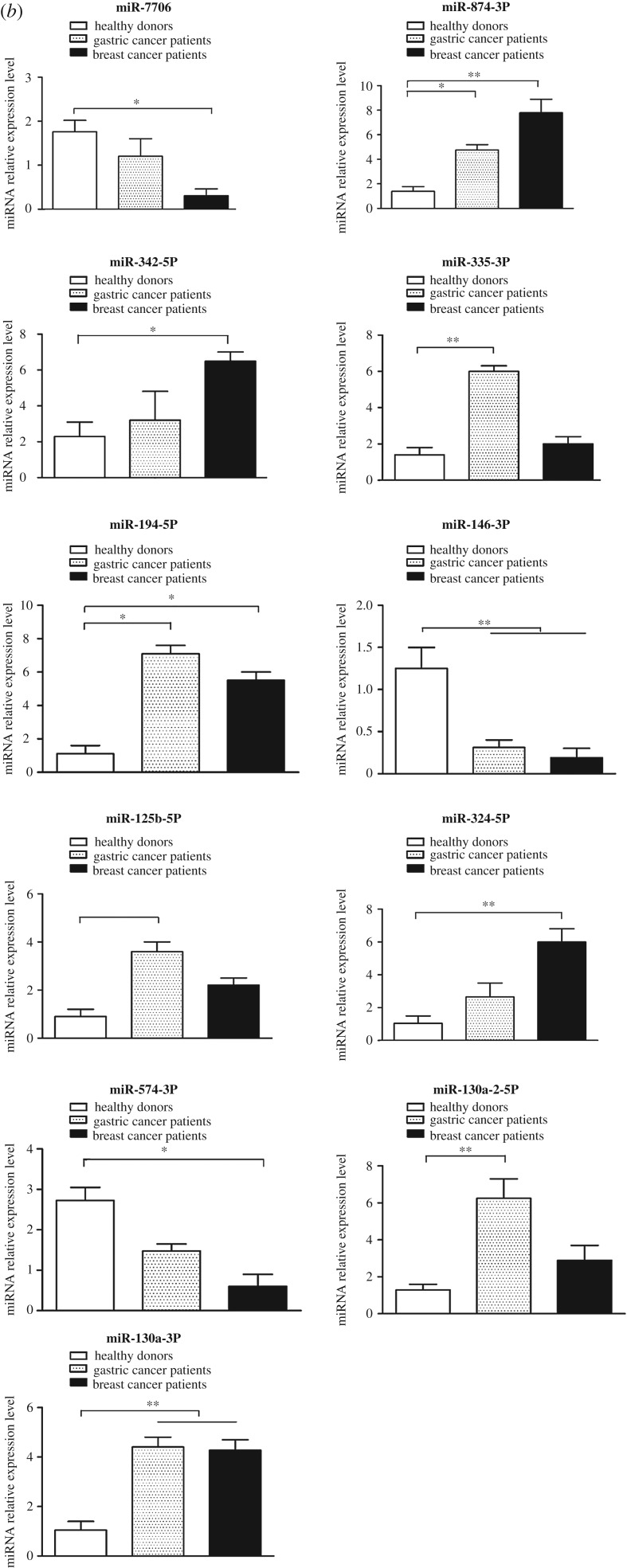
The miRNAs of monocytes associated with cancers. (*a*) Heatmap of differentially expressed miRNAs in monocytes of cancer patients and healthy donors. The numbers on the right indicated the log10 of the number of miRNA copies. (*b*) The expression profiles of selected miRNAs in monocytes of cancer patients and healthy donors. Total RNAs extracted from monocytes were subjected to quantitative real-time PCR to determine the expression levels of miRNAs. U6 RNA was used as a loading control. All the numerical data are represented as the mean ± standard deviation of triplicate assays.

To reveal the miRNAs associated with cancers, the expression patterns of miRNAs from the transformed monocytes of breast cancer patients, gastric cancer patients and healthy donors were compared. The results demonstrated that 74 miRNAs were significantly upregulated in both breast and gastric cancer patients compared with healthy donors, while 46 miRNAs were significantly downregulated in cancers (figure 1*a* and table 4), suggesting that these 120 miRNAs were associated with cancers. By comparison with the healthy donors, there were 10 or 35 miRNAs upregulated only in breast cancer samples or only in gastric cancer samples and nine or five miRNAs downregulated only in breast cancer patients or only in gastric cancer patients (figure 1*a* and table 4).

**Table 4.** The differentially expressed miRNAs in monocytes from patients with gastric cancer and in patients with breast cancer. n.a., not applicable. *p-*value indicates the statistical significance of the miRNA expression levels in samples from patients with gastric cancer versus those from patients with breast cancer.

name	log2 fold change	chromosomal location	validated in ref.	*p-*value
hsa-miR-539-5p	3.2818	14q32.31	[30]	0.00180
hsa-miR-3134	2.6968	3p25.1	[31]	0.00311
hsa-miR-376a-5p	2.6968	14q32.31	[30]	0.00311
hsa-miR-548e-5p	2.6968	10q25.2	[32]	0.00311
hsa-miR-933	2.4338	2q31.1	[33]	0.00377
hsa-miR-9903-1	2.4338	n.a.	n.a.	0.00077
hsa-miR-959	2.3342	n.a.	n.a.	0.00148
hsa-miR-548ap-3p	2.1993	15q25.3	[34]	0.00121
hsa-miR-1256	2.1118	1p36.12	[32]	0.00461
hsa-miR-1538	2.1118	16q22.1	[35]	0.00461
hsa-miR-877-3p	2.1118	6p21.33	[30]	3.45×10^−5^
hsa-miR-31-3p	−2.0036	9p21.3	[30]	0.00355
hsa-miR-378g	−2.0036	1p21.3	[36]	0.00355
hsa-miR-5091	−2.1106	4p15.33	[37]	1.22×10^−10^
hsa-miR-138-5p	−2.1361	16q13	[30]	0.00239
hsa-miR-4781-3p	−2.2101	1p32.3	[38]	0.00215
hsa-miR-195-3p	−2.2101	17p13.1	[30]	0.00035
hsa-miR-365a-5p	−2.2101	16p13.12	[30]	0.00283
hsa-miR-545-5p	−2.2101	Xq13.2	[30]	2.25×10^−7^
hsa-miR-6734-5p	−2.2101	1p34.2	[39]	0.00035
hsa-miR-6802-3p	−2.2101	19q13.42	[39]	0.00535
hsa-miR-3176	−2.6955	16p13.3	[31]	0.00098
hsa-miR-4742-3p	−2.6955	1q42.11	[38]	0.00008

To validate the differentially expressed miRNAs, the expression profiles of 11 randomly selected miRNAs in the transformed monocytes from cancer patients and healthy donors were examined. The results of quantitative real-time PCR showed that the expression patterns of the 11 selected miRNAs were consistent with the data of miRNA sequencing (figure 1*b*).

### 3.4. Involvement of miR-150-5p of THP-1 monocytes in tumorigenesis

**Figure 2.**
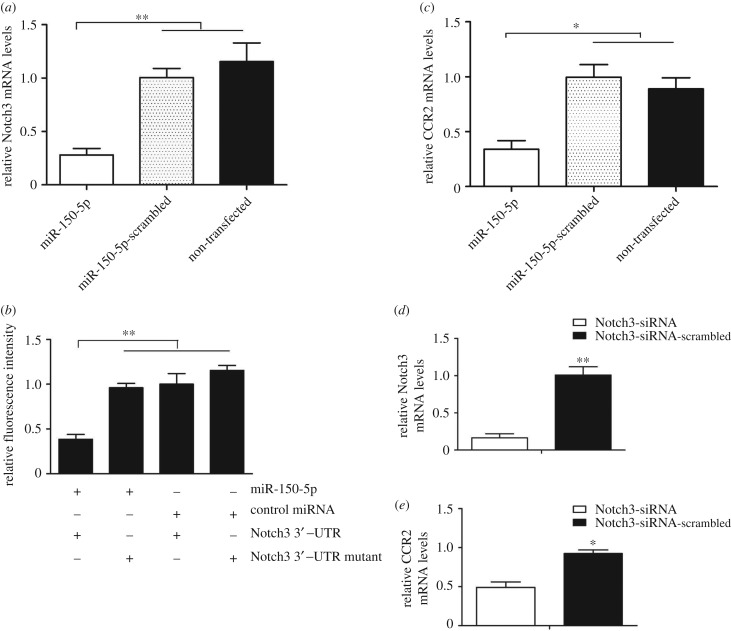
Involvement of miR-150-5p of THP-1 monocytes in tumorigenesis. (*a*) Interaction between *Notch3* and miR-150-5p. THP-1 cells were transfected with miR-150-5p or miR-150-5p-scrambled, followed by the examination of CCR2 mRNA using quantitative real-time PCR. As a control, non-transfected cells were included in the assays. (*b*) The direct interaction between *Notch3* and miR-150-5p. THP-1 cells were co-transfected with miR-150-5p and a luciferase reporter fused with Notch3 3′-UTR. At 24 h after transfection, the firefly and *Renilla* luciferase activities were analysed. Control miRNA and Notch3 3′-UTR mutant were included in the co-transfections as controls. (*c*) Influence of miR-150-5p on CCR2 expression. THP-1 cells were transfected with miR-150-5p or miR-150-5p-scrambled and then quantitative real-time PCR was conducted to detect the mRNA contents of CCR2. Non-transfected cells were used as a control. (*d*) Silencing of *Notch3* in THP-1 cells. Notch3-siRNA was transfected into THP-1 cells to silence the expression of *Notch3*. Notch3-siRNA-scrambled was used as a control. At 24 h after transfection, the expression of *Notch3* was evaluated with quantitative real-time PCR. (*e*) Impact of *Notch3* silencing on the expression of CCR2. In all panels, the statistical significance between treatments is indicated with asterisks (*p < 0.05; **p < 0.01).

As an example, miR-150-5p, a miRNA differentially expressed in monocytes of breast cancer patients and healthy controls, was characterized in THP-1 monocytes to explore the role of cancer-associated miRNAs in tumorigenesis. The target gene prediction analysis showed that *Notch3* was a potential target gene of miR-150-5p. It was found that the transfection of miR-150-5p significantly decreased *Notch3* expression in THP cells compared with the cells transfected with miR-150-5P-scrambled and the non-transfected cells (figure 2*a*), confirming that *Notch3* was the target gene of miR-150-5p.

To explore the direct interaction between miR-150-5p and *Notch3*, dual-luciferase reporter assays were conducted in THP-1 cells. Our results showed that the luciferase activity of the cells transfected with miR-150-5p and Notch3 3′-UTR was significantly decreased compared with the controls (figure 2*b*). When the region complementary to the seed sequence of miR-150-5p was mutated, the luciferase activity of the cells co-transfected with miR-150-5p and Notch3 3′-UTR mutant was consistent with the controls (figure 2*b*). These data showed that miR-150-5p specifically targets the *Notch3* gene.

As reported, the CCR2 protein of monocytes can promote breast cancer metastasis through the Notch 3 pathway [40]. Therefore, an impact of miR-150-5p on CCR2 expression was explored in this study. The results showed that the CCR2 expression was significantly downregulated when the THP-1 cells were transfected with miR-150-5p in comparison with miR-150-5p-scrambled-transfected and non-transfected cells (figure 2*c*).

To evaluate the effects of *Notch3* downregulation mediated by miR-150-5p on the CCR2 expression, Notch 3-specific siRNA (Notch3-siRNA) was used to silence the *Notch3* expression (figure 2*d*). The results revealed that the CCR2 expression was dramatically reduced when *Notch3* was silenced (figure 2*e*). These data indicated that miR-150-5p downregulated CCR2 expression by suppressing *Notch3* expression.

Taken together, these findings demonstrated that miR-150-5p downregulated the CCR2 expression in monocytes by targeting *Notch3*, suggesting its involvement in tumorigenesis.

### 3.5. Pathways mediated by cancer-associated miRNAs

**Figure 3.**
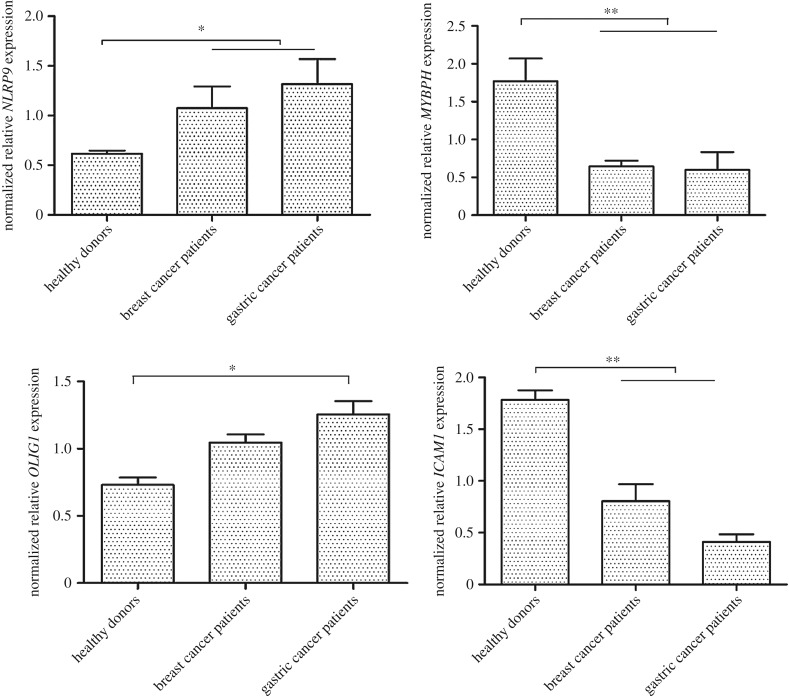
The expression patterns of target genes in monocytes of cancer patients and healthy donors. Total RNAs were extracted from monocytes of cancer patients and healthy donors. The expression levels of target genes, selected at random, were examined with quantitative real-time PCR. Among the target genes, *NLRP9*, *MYBPH*, *OLIG1*and *ICAM1* were predicted to be targeted by miR-146a-3p, miR-130a-3p, miR-7706 and miR-874-3p, respectively. The differences between treatments are indicated with asterisks (*p < 0.05; **p < 0.01).

To reveal the potential pathways of cancer-associated miRNAs, the genes targeted by the 120 miRNAs that were simultaneously upregulated or downregulated in breast and gastric cancer patients, were predicted. The results of miRanda, PITA and RNAhybrid analysis showed that 36 of the 120 cancer-associated miRNAs had overlapping target genes. Most of the target genes of the miRNAs were involved in tumorigenesis. They could be divided into three categories, including the genes regulating proliferation of cancer cells and tumour metastasis, as well as the genes used as prognostic factors or biomarkers for patients with cancers. The quantitative real-time PCR data indicated that the expression patterns of the miRNA target genes, selected at random, were consistent with the sequencing analysis (figure 3). These findings suggested that the 36 miRNAs were associated with cancers by targeting their target genes.

**Figure 4.**
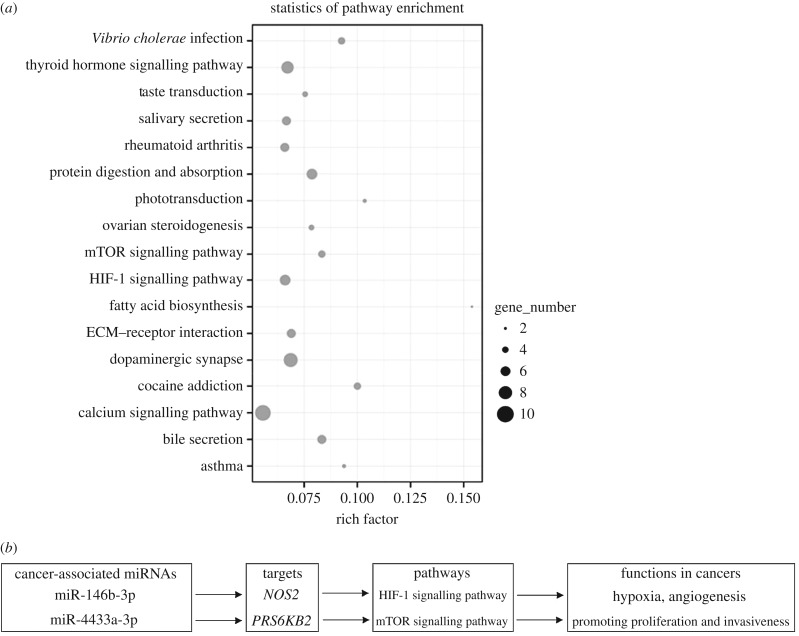
Pathways mediated by cancer-associated miRNAs. (*a*) Statistics of the most enriched pathways from KEGG analysis. The target genes of cancer-associated miRNAs were used for this analysis. The enriched gene number in each corresponding biological pathway is reflected by the size of the grey cycle. (*b*) Pathways mediated by miR-146b-3p and miR-4433a-3p in cancers.

For an overview of the protein networks and biological functions related to the cancer-associated miRNAs, the inferred target genes were collected for KEGG pathway analysis. The results showed that many target genes were involved in the signalling pathways of cancer progression, such as the mTOR signalling pathway [41,42], the HIF-1 signalling pathway [43,44] and the calcium signalling pathway [45] (figure 4*a*). Thus, the cancer-associated miRNAs might play important roles in cancer development. For example, miR-146b-3p and miR-4433a-3p could, respectively, target *NOS2* (nitric oxide synthase 2) and *RPS6KB2* (ribosomal protein S6 kinase B2) of the HIF-1 signalling pathway and the mTOR signalling pathway, leading to the proliferation and invasion of cancers [46,47] (figure 4*b*).

## 4. Discussion

Cancer is one of the major causes of human death with increasing incidence and mortality worldwide. At present, many genes or proteins differentially expressed between normal and corresponding tumour tissues have been identified for the diagnosis and treatment of human cancers [48–50]. However, knowledge of these protein or gene biomarkers seems insufficient for cancer therapy. Extensive investigations of miRNAs have revealed that they participate in the complicated regulatory networks linking genes with biological processes of cancer, thus forming a new level for the diagnosis and treatment of cancers. In breast cancer, some miRNAs, such as miR-126 and miR-335, can suppress tumour growth and proliferation [51]. In gastric cancer cells, the abnormally upregulated miRNA clusters (miR-106b–93–25 and miR-222–221) promote tumour growth by targeting the Cip/Kip family members of Cdk inhibitors (p57Kip2, p21Cip1 and p27Kip1) [52]. It has been reported that the differentially expressed miRNAs can act as potential biomarkers for cancer diagnosis and prognosis [53,54]. The level of Let-7a, a diagnosis and prognosis biomarker used in both breast and gastric cancers, is significantly lower in patients than in healthy donors [53,54]. Thus, miRNAs have attracted increasing attention in recent years for their roles in tumorigenesis. Although a spectrum of differentially expressed miRNAs associated with cancer development embodies biomarkers for cancer diagnosis [7,8], the information about the abnormal expressions of miRNAs in monocytes, important cells in tumour immunity, is very limited. This study revealed that 120 miRNAs were upregulated or downregulated in monocytes of patients with breast or gastric cancers compared with healthy donors. It has also been found that the function of monocytes can be changed when the surrounding normal microenvironment is transformed into a tumour microenvironment [55]. In this investigation, the differentially expressed miRNAs from the transformed monocytes of cancer patients might play important roles in cancers. In this context, our findings provide novel clues to explore the relationship between miRNAs and cancers.

Cancer progression is a very complicated process involving various kinds of gene expression, which can be regulated by miRNAs. The 120 cancer-associated miRNAs revealed in this study might act as regulators controlling proliferation and metastasis of tumours. For example, miR-146b-3p might function to regulate the proliferation of cancers by targeting *NOS2* gene in the HIF-1 signalling pathway. NOS2 can regulate cancer proliferation via increasing the stability of HIF-1a [46]. *RPS6KB2*, the target gene of miR-4433a-3p, participates in cancer progression through the mTOR signalling pathway [47]. MiR-106a-5p, a cancer-associated miRNA revealed in the present study, can inhibit proliferation and migration of astrocytoma cells and promote apoptosis by targeting *FASTK* [56]. As a tumour suppressor, miR-146a suppresses the anchorage-independent growth, migration and invasion of prostate cancer cells by directly targeting the *Rac1* gene [57]. As is well known, miRNAs can serve as biomarkers for the diagnosis and prognosis of cancers. Two cancer-associated miRNAs (miR-144-5p and miR-19b-3p) revealed in this investigation have been found to be potential prognostic markers in bladder cancer and gastric cancer [58]. Therefore, it could be speculated that the 120 cancer-associated miRNAs found in this study provide important clues about the mechanisms of cancer progression. The 120 cancer-associated miRNAs merit further characterization using more samples from cancer patients.

## 5. Conclusion

Based on the characterization of the differentially expressed miRNAs in peripheral blood monocytes of patients with gastric or breast cancers and healthy donors, 120 cancer-associated miRNAs have been revealed. Among these miRNAs, 38 miRNAs are novel. The signature miRNAs are predicted to be involved in the pathways associated with cancer progression, suggesting that they play important roles in cancers.

## Data accessibility

Raw sequence data have been deposited in the Short Read Archive (SRA) database of NCBI with the accession number: SRP080085.
